# The Effects of the Combination of Oral Lactoferrin and Iron Injection on Iron Homestasis, Antioxidative Abilities and Cytokines Activities of Suckling Piglets

**DOI:** 10.3390/ani9070438

**Published:** 2019-07-11

**Authors:** Ping Hu, Daoyuan Zhao, Fangzhou Zhao, Jing Wang, Weiyun Zhu

**Affiliations:** 1 National Center for International Research on Animal Gut Nutrition, Jiangsu Key Laboratory of Gastrointestinal Nutrition and Animal Health, Laboratory of Gastrointestinal Microbiology, National Experimental Teaching Demonstration Center of Animal Science, College of Animal Science and Technology, Nanjing Agricultural University, Nanjing 210095, China; 2 College of Science, Nanjing Agricultural University, Nanjing 210095, China

**Keywords:** lactoferrin, iron injection, iron level, antioxidant ability, cytokines, suckling piglets

## Abstract

**Simple Summary:**

Previous studies have shown that an iron injection alleviates iron deficiency in suckling piglets, but decreases the fractional iron absorption and causes acute poisoning with poor efficiency of antioxidant system, and has other undesirable side effects. Lactoferrin is a critical regulator of iron absorption and oxidative stress. The present results showed that the combination of oral lactoferrin and iron injection is a more effective method to improve iron level, enhance antioxidant ability and modulate the cytokine activity in the suckling piglets.

**Abstract:**

Iron deficiency is considered a common nutritional problem for suckling piglets. The aim of this study was to evaluate the effects of the combination of oral lactoferrin and iron injection on iron levels, antioxidant ability and cytokine activity in suckling piglets. A total of sixty suckling piglets taken from six sows (10 piglets per litter) with a similar parity were chosen. The lactoferrin (LF) group was orally administrated with lactoferrin solution (0.5 g/kg body weight per day) for a week, the CON group was orally administrated with the same dose of physiological saline. Each piglet (all groups) was given 100 mg of iron dextran (FeDex) by intramuscular injection at the third day of age. Six piglets (n = 6) from each group were euthanized on days 8 and 21. The oral lactoferrin improved the iron level of suckling piglets by increasing the concentrations of serum hemoglobin and hepatic iron on day 8. Gene expression of lactoferrin receptor (LFR) was significantly increased in the LF group piglets on day 8, while duodenal protein expression of the divalent metal transporter 1 (DMT1) was significantly reduced in the LF group on day 8. In addition, oral lactoferrin enhanced serum T-AOC activities and duodenal SOD activities on day 21. The LF piglets had a significantly increased serum concentration of IL-10 on day 8. These results indicated that a combination of oral lactoferrin and iron injection is a more effective method of improving the iron level by up-regulating the expression of the LFR gene, enhancing the antioxidant ability and modulating the cytokine activity in the suckling piglets.

## 1. Introduction

Iron deficiency is considered a common nutritional problem for young animals in intensive farming, which contributes to the dysfunction of internal environment of young animals and induces the destruction of oxidative balance and immune function of young animals [1,2]. For suckling piglets, about 25 mg/day of iron is needed for erythropoiesis and other functions. Sow milk is a poor source of iron and provides suckling piglets with only 1 mg of iron per day [3,4]. It is impossible for suckling piglets to obtain sufficient iron from sow’s milk alone. Therefore, intramuscular injection of exogenous iron has been extensively used to prevent iron deficiency of suckling piglets in the swine industry [5]. However, intramuscular injection of a large amount of iron dextran to suckling piglets may easily perturb the control of systemic iron metabolic processes and heighten iron overload in tissues [6,7]. In addition, some investigations revealed that the administration of iron-dextran in suckling piglets could decrease the fractional iron absorption and cause acute poisoning with poor efficiency of the antioxidant system and undesirable side effects [8,9]. These findings suggest that injecting an iron supplement alone may not be an effective strategy to maintain the balance between the iron levels and body health in young animals.

Lactoferrin, an iron-binging multifunctional cationic glycoprotein, is a vital element of host physiology [10]. It is noteworthy to know that the administration of lactoferrin influences iron absorption and metabolism [11]. On one hand, lactoferrin promotes iron absorption to satisfy the iron demand when the body is in an iron-deficient state. On the other hand, lactoferrin chelates with iron to prevent the body from being damaged by excessive iron through reducing the absorption of iron when the needs of iron metabolism are met [12]. Previous research demonstrated that intraperitoneal injection of lactoferrin led to an increase in hemoglobin (Hb) level in an acute anemia rat model [13]. In addition, the supplement of lactoferrin alleviated the iron deficiency symptoms though increasing the levels of Hb and total serum iron (TSI) in pregnant women, even though the concentration of iron supplemented as lactoferrin is far from sufficing the daily iron requirement [14]. Therefore, the lactoferrin efficacy in curing iron deficiency may not relate with direct iron supplement, but through the different mechanisms involving iron absorption. Lactoferrin supplementation is not only an effective way to relieve iron deficiency symptoms, but also a useful method to enhance antioxidant levels and inhibit the inflammatory responses, which are associated with intestinal health [15,16]. Most studies have investigated the impacts of lactoferrin on iron homeostasis in adult humans or weaned animals, but there is limited literature that focuses on the effects of lactoferrin iron homeostasis of the suckling animals.

Hence, we hypothesize that the combination of oral lactoferrin and iron injection is an effective method to prevent iron deficiency and maintain the intestinal health of suckling piglets. The present study was conducted to explore the effects of the combination of oral lactoferrin and iron injection on the iron levels, the expression of genes and proteins involved in iron absorption, the antioxidant ability and the cytokine activity of suckling piglets.

## 2. Materials and Methods

The usage of animals in this experiment were consistent with the Chinese Guidelines for Animal Welfare and were approved by the Animal Care and Use Committee of Nanjing Agricultural University (Nanjing, Jiangsu province, China). The license number is SYXK-2017-0027 with a period of validity to 19 June 2022.

### 2.1. Animals and Samples

The animal experiment was performed in a commercial farm in Jiangsu Province, China. A total of 60 suckling piglets (Duroc × Landrace × Yokshire) from six sows (10 piglets per litter) with similar parity (2–3 parities) were chosen in this study. To avoid maternal differences, piglets in each litter were randomly divided into the control (CON) group and the lactoferrin (LF) group according to the body weight (1.51 ± 0.1 kg). Each group therefore consisted six replicates (n = 6) with five newborn piglets. The piglets in the LF group were administrated with 8–12 mL lactoferrin solution (The lactoferrin dissolved in water to reach 30–40 mg/mL concentration) three times per day at 09:00, 14:00 and 19:00 (0.5 g/kg body weight per day) (Ingradia, France) from the age of 1 to 7 days, and the piglets in the CON group were orally administrated with the same dose of physiological saline. In addition, each piglet was given 100 mg of iron dextran (FeDex) (ZHINONG HUIMU, Shandong, China) by intramuscular injection on the third day of age. All piglets were weaned on day 21. During the experimental period (21 days), piglets had no access to creep feed and the sow’s milk was their sole source of nutrients. On day 8 and day 21, one piglet from each litter (six piglets from each group, n = 6) was slaughtered with a jugular vein injection of 4% sodium pentobarbital solution (40 mg/kg body weight). Blood samples were collected from the jugular vein and serum was obtained by centrifuging at 3000× *g* for 15 min at 4 °C and then stored at −80 °C until analysis. The abdomen was opened and the segments (stomach, duodenum, jejunum, ileum, cecum, and colon) were identified and ligated before separation. The digesta in the terminal ileum was collected and mixed for further analysis. The samples of the liver, spleen, kidney, muscle, duodenum, jejunum and ileum were collected and stored at −80 °C until assay.

### 2.2. Iron Parameters Analysis

The concentrations of hemoglobin in the serum were determined using an automated hematology analyzer (Sysmex K-1000D, Sysmex Inc., Kobe, Japan). The concentrations of serum iron and total iron binding capacity (TIBC) in the serum were measured using commercial kits (serum iron assay kit, No: A039-1; TIBC assay kit, No: A040, Jiancheng, China). The concentrations of hepcidin in the serum and liver were measured by using a hepcidin ELISA kit (NO: H252, Jiancheng, China). Iron concentrations in the tissues and digesta were measured by inductively coupled plasma atomic emission spectroscopy (ICP-AES) according to the procedures described previously [17]. Briefly, 0.25 g of tissues or digesta (dried samples, the tissues and digesta were put in the freeze dryer for 24 h), 5 mL of 65% HNO_3_ and 2 mL of 30% H_2_O_2_ were mixed in a polytetrafluoroethylene (PTFE) tube. Then, the samples were digested in a microwave sample digestion system (MARS 6, CEM Corporation, Matthews, NC, USA). Afterwards, the PTFE tubes were kept in a heater to remove acid. Five percent of HNO_3_ was used to dissolve the tube residues in the PTFE tubes. Finally, the prepared solution was stored in a refrigerator at 4 °C until analysis.

### 2.3. Gene Expression Analysis

Total RNA was extracted from the duodenum using the Trizol Reagent (Incitrogen, Carlsbad, CA, USA). Total RNA (1 μg) was revered-transcribed to cDNA using PrimeScript RT reagent Kit with gDNA eraser (TaKaRa Biotechnology, Dalian, China) according to manufacturer’s instructions. The primers used for iron related genes (duodenal cytochrome b (Dcytb), transferrin (TF), transferrin receptor 1 (TFR 1), transferrin receptor 2 (TFR 2) and lactoferrin receptor (LFR) are shown in Table 1. The mRNA levels were normalized to the expression of β-actin gene, and the relative expression levels were calculated by using the 2^−ΔΔCt^ method (ΔΔCt = (Ct_Targe_t − Ct _β-actin_)treatment − (Ct_Target_ − Ct_β-actin_)_control_) [18].

### 2.4. Protein Extraction and Western Blot Analysis

Total protein was extracted from frozen duodenal mucosa using Radio Immunoprecipitation Assay lysis buffer (Beyotime Institute of Biotechnology, Shanghai, China), protease inhibitors (Beyotime Institute of Biotechnology, Shanghai, China), and phosphatase inhibitors (Sigma-Aldrich, St. Louis, MO, USA). Protein concentrations were measured using a BCA Protein Assay Kit (Beyotime Institute of Biotechnology, Shanghai, China). The primary antibodies were used as follows: anti-DMT1 (1:1000, 20507-1-AP, Proteintech, Wuhan, China), anti-FPN (1:1000, 26601-1-AP, Proteintech, Wuhan, China) and anti-beta Actin (1:2000, Abcam, Cambridge, MA, USA). The band density of target proteins was normalized with that of the β-actin protein. Membranes were washed, incubated with secondary antibody, visualized, and band densities were semiquantified as previously described [19].

### 2.5. Antioxidant Status Analysis

Intestinal mucosa (0.1 g, n = 6) was homogenized in ice-cold PBS and then centrifuged at 3500× *g* at 4 °C for 15 min, and the supernatant was stored at −80 °C. Protein concentration was determined using a BCA Protein Assay Kit (Beyotime Institute of Biotechnology, Shanghai, China). The serum and tissue levels of glutathione peroxidase (GSH-px), superoxide diamutase (SOD), total anti-oxidation capacity (T-AOC) and malondiadehyde (MDA) were measured by the commercial kits (GSH-PX, A005; SOD, A001-1; T-AOC, A015; MDA, A003-1) (Jiancheng Bioengineering Institute of Nanjing, Nanjing, China) according to the manufacturer’s instructions. 

### 2.6. Serum Cytokines Analyses

The serum levels of IL-1α, IL-1β, IL-10, and TNF-α were measured by enzyme-linked immunosorbent assay (ELISA) kits purchased from R & D Systems (Shanghai, China). The detection limits were 10.0 pg/mL for IL-1α, 10.0 pg/mL for IL-1β, 8.0 pg/mL for IL-10 and 1.0 pg/mL for TNF-α, respectively.

### 2.7. Statistical Analysis

Data were analyzed by SPSS 22.0 and expressed as the mean with their standard errors. The data were analyzed as a 2 × 2 factorial with the general linear model procedures of the Statistical Analysis package. The model included the fixed effects of diet (with or without LF), age (on day 8 or day 21) and their interaction. The piglet was the experimental unit. All data were analyzed by two-way ANOVA, and differences were considered significant at *p* < 0.05. When a significant effect of the interaction (*p* < 0.05) between diet and age was observed, the data were further analyzed by one-way ANOVA with Duncan’s post hoc test. A value of *p* < 0.05 was considered to be statistically significant, while *p* values between 0.05 and 0.10 were considered as a tendency.

## 3. Results

### 3.1. Effects of Lactoferrin on Serum Iron Parameters of Suckling Piglets

The iron levels in the serum are shown in Table 2. Hemoglobin concentration was significantly affected by diet, age and their interaction (*p* < 0.05). Hemoglobin concentration in the LF piglets was higher (*p* < 0.05) than that in the CON piglets on day 8 and was significantly increased on day 8 compared to day 21 (*p* < 0.05). In addition, total iron binding capacity (TIBC) was not affected by diet, age and their interactions (*p* > 0.05).

### 3.2. Effects of Lactoferrin on Iron Levels in the Tissues and Digesta of Suckling Piglets

The iron levels in the tissues and digesta are shown in Table 3. Hepatic iron concentration was statistically influenced by diet (*p* < 0.05), and the hepatic iron concentration in LF piglets was higher compared with that in the CON piglets (*p* < 0.05). Spleen iron concentration was significantly affected by age (*p* < 0.05), and spleen iron concentration was higher on day 8 than that on day 21 (*p* < 0.05). Furthermore, the iron concentration in the ileal digesta was significantly affected by diet and age (*p* < 0.05), and the digesta iron concentration was statistically decreased in the LF piglets compared with that in the CON piglets (*p* < 0.05). For kidney, muscle, duodenum, jejunum and ileum, iron levels were influenced by age and were higher on day 8 than those on day 21 (*p* < 0.05).

### 3.3. Effects of Lactoferrin on the Expressions of Gene and Protein Related to Iron Metabolism in the Duodenum of Suckling Piglets

**Figure 1:**
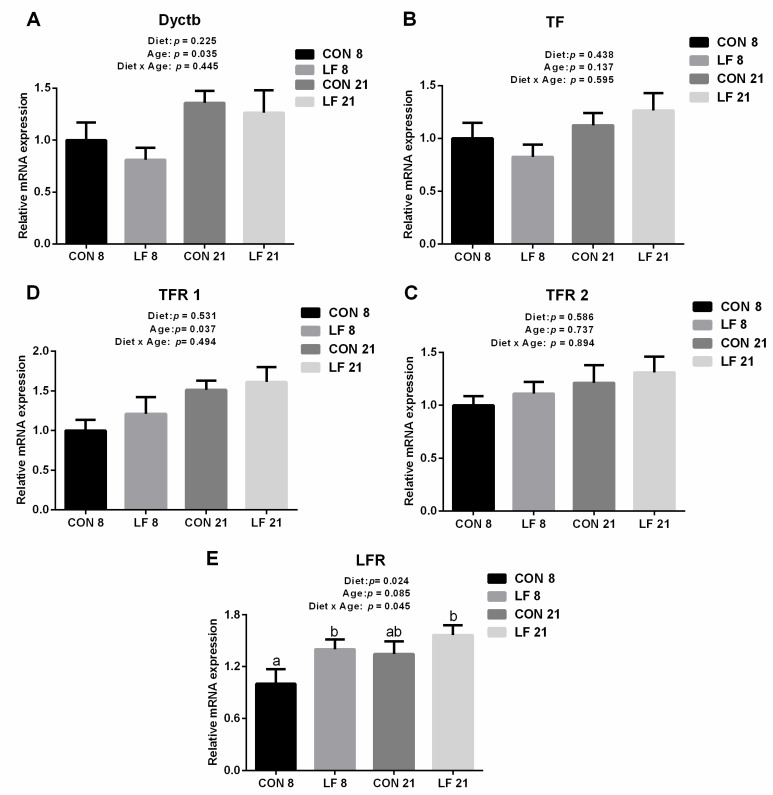
Effects of lactoferrin on gene expressions related to iron metabolism in the duodenum of suckling piglets. (**A**) duodenal cytochrome b (Dcytb) (**B**) transferrin (TF) (**C**) transferrin receptor 1 (TFR 1) (**D**) transferrin receptor 2 (TFR 2) (**E**) lactoferrin receptor (LFR). Values are means, standard errors represented by vertical bars, n = 6 per treatment. ^a-b^ Different letters mean significantly different for different groups at *p* < 0.05. CON 8, control group on day 8; LF 8, Lactoferrin group on day 8; CON 21, Control group on day 21; LF 21, Lactoferrin group on day 21.

**Figure 2:**
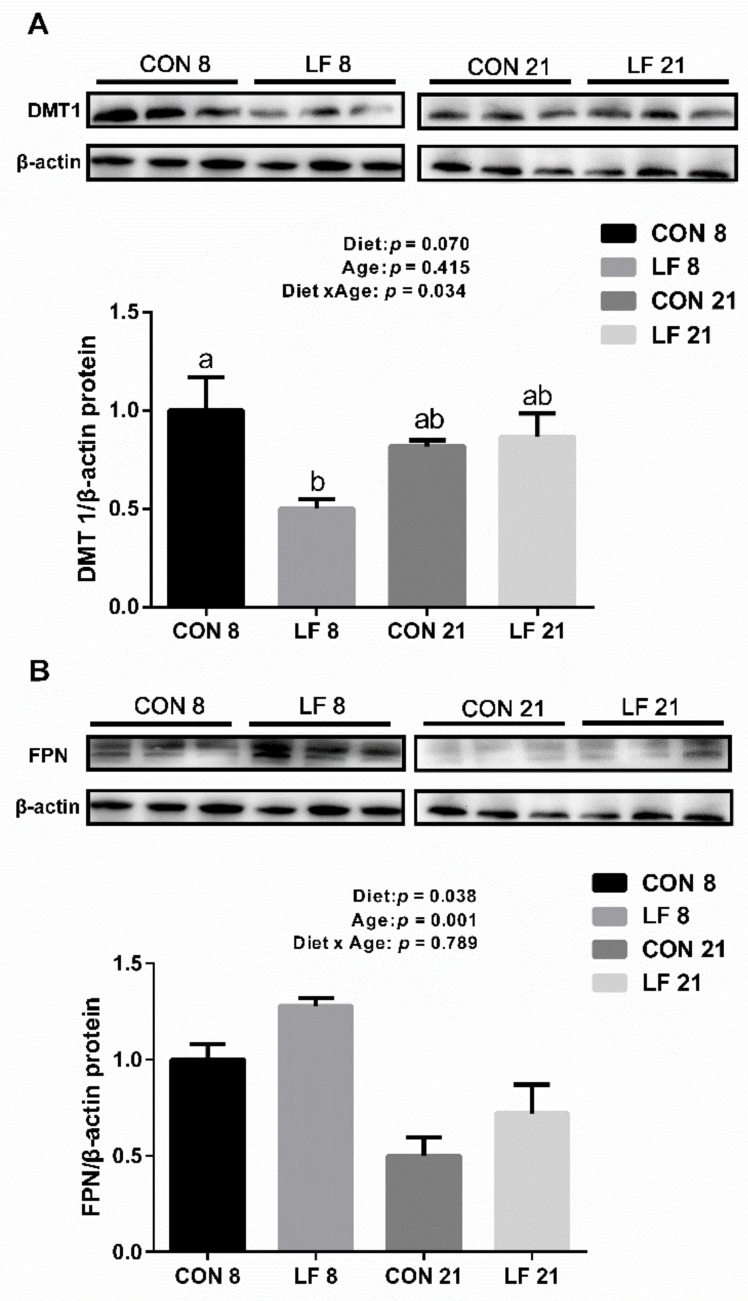
Effects of lactoferrin on protein expressions related to iron metabolism in the duodenum of suckling piglets. (**A**) divalent metal transporter 1 (DMT 1) (**B**) ferroportin (FPN). Values are means, standard errors represented by vertical bars, n = 6 per treatment. ^a-b^ Different letters mean significantly different for different groups at *p* < 0.05. Control group on day 8 (CON 8); Lactoferrin group on day 8 (LF 8); Control group on day 21 (CON 21); Lactoferrin group on day 21 (LF 21).

The expressions of gene and protein related iron metabolism are presented in Figure 1 and Figure 2, respectively. At the gene level (Figure 1), the gene expressions of Dcytb and TFR 1 were significantly affected by age, and the gene expressions of Dcytb and TFR 1 were higher on day 21 than those on day 8 (*p* < 0.05). In addition, the gene expressions of LFR were statistically affected by diet and the interaction between diet and age (*p* < 0.05), with higher gene expressions in the LF piglets than those in the CON piglets on day 8 (*p* < 0.05). At the protein level, the protein expressions of DMT1 were significantly affected by the interaction between diet and age (*p* < 0.05), and the protein expressions of DMT1 were statistically down-regulated in the LF piglets compared with the CON piglets on day 8 (*p* < 0.05). The protein expressions of FPN were significantly affected by diet and age (*p* < 0.05), with higher expressions in the LF piglets than those in the CON piglets (*p* < 0.05).

### 3.4. Effects of Lactoferrin on Hepcidin Levels in the Serum and Liver of Suckling Piglets

**Figure 3:**
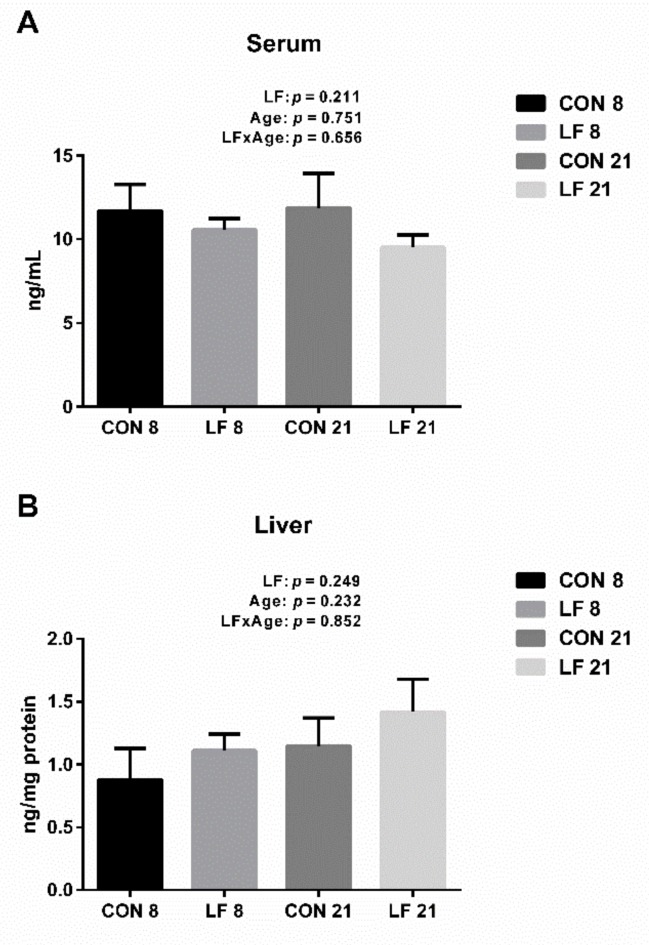
Effects of lactoferrin on hepcidin levels in the serum (**A**) and liver (**B**) of suckling piglets. Values are means, standard errors represented by vertical bars, n = 6 per treatment. Control group on day 8 (CON 8); Lactoferrin group on day 8 (LF 8); Control group on day 21 (CON 21); Lactoferrin group on day 21 (LF 21).

The hepcidin levels in the serum and liver are shown in the Figure 3. The hepcidin levels in the serum and liver were not significantly affected by diet, age and their interaction (*p* > 0.1). Hence, there was no significant difference on the levels of hepcidin among all groups (*p* > 0.1).

### 3.5. Effects of Lactoferrin on Antioxidant Ability in the Serum and Duodenum of Suckling Piglets

The antioxidant levels in the serum and duodenum are presented in the Table 4 and Table 5, respectively. In the serum (Table 4), T-AOC activity was significantly affected by diet and the interaction between diet and age (*p* < 0.05), and T-AOC activity was significantly increased in the LF piglets compared with the CON piglets on day 21 (*p* < 0.05). GSH-px activity was affected by diet and age (*p* < 0.05), and GSH-px activity was higher in the LF piglets than that in the CON piglets (*p* < 0.05). For the duodenum (Table 5), SOD activity was significantly affected by the interactions between diet and age (*p* < 0.05), with higher SOD activity in the LF piglets than that in the CON piglets on day 21 (*p* < 0.05). T-AOC activity in the duodenum was significantly influenced by age and the interaction between diet and age (*p* < 0.05). MDA activity was statistically affected by age, and MDA activity was higher on day 8 than that on day 21 (*p* < 0.05). In addition, GSH-px activity was significantly affected by diet (*p* < 0.05) and were higher in the LF piglets than that in the CON piglets (*p* < 0.05).

### 3.6. Effects of Lactoferrin on Cytokines Levels in the Serum of Suckling Piglets

**Figure 4:**
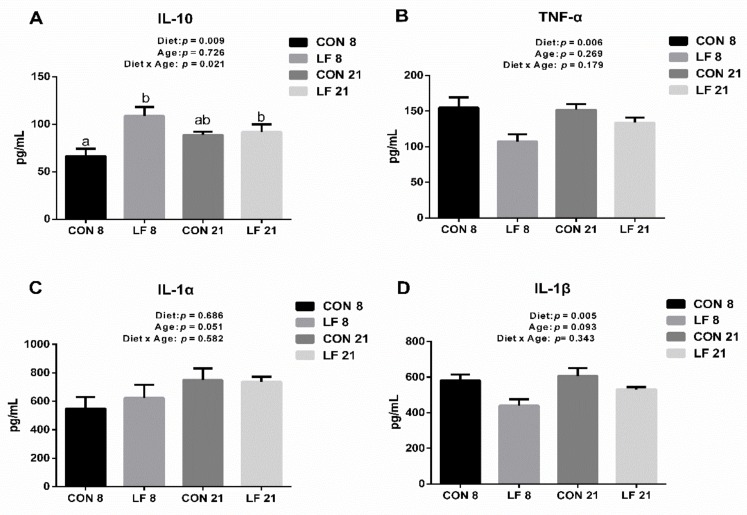
Effects of lactoferrin on cytokines levels in the serum of suckling piglets. (**A**) IL-10, (**B**) TNF-α, (**C**) IL-1α, (**D**) IL-1β. Values are means, standard errors represented by vertical bars, n = 6 per treatment. ^a-b^ Different letters mean significantly different for different groups at *p* < 0.05. Control group on day 8 (CON 8); Lactoferrin group on day 8 (LF 8); Control group on day 21 (CON 21); Lactoferrin group on day 21 (LF 21).

The levels of cytokines in the serum are shown in the Figure 4. The IL-10 concentration was significantly affected by diet and the interaction between diet and age (*p* < 0.05), and the IL-10 concentration in the LF piglets was higher than that in the CON piglets on day 8 (*p* < 0.05). The concentration of TNF-α was statistically influenced by diet and were lower in the LF piglets than that in the CON piglets (*p* < 0.05). Furthermore, the concentration of IL-1β was significantly affected by diet (*p* < 0.05), and the concentration of IL-1β was statistically decreased in the LF piglets. No statistical difference on IL-1α concentrations was observed among all groups (*p* > 0.05).

## 4. Discussion

Iron deficiency is considered a common nutritional problem for suckling piglets due to the rapid growth and the increasing number of red blood cells of suckling piglets [20]. Iron injection is the most common method to prevent iron deficiency, but causes undesirable side effects in suckling piglets. We have studied the effects of the combination of oral lactoferrin and iron injection on iron homeostasis and intestinal health of suckling piglets. The colostrum is the best source of nutrition for suckling piglets. Hence, the dose of lactoferrin used in the present study was determined based on the assumption that a neonatal piglet (1.5 kg body weight) consumes 400–700 mL colostrum/day, with a mean LF concentration of 1.6 g/L, which is approximately equal to 0.5 g/kg body weight [21,22]. Consequently, the dose of lactoferrin was 0.5 g/kg body weight in this study. In the current study, the concentration of serum hemoglobin and hepatic iron was higher in the LF piglets than that in the CON piglets on day 8, suggesting that the combination of oral lactoferrin and iron injection may be a more effective method to improve iron level for suckling piglets [4]. These results were consistent with the finding of Fernández-Menéndez et al., who demonstrated that the combination of lactoferrin and iron salt is able to enhance iron level and absorption for young animals [23]. It should be noted that a lower concentration of iron was detected in the ileal digesta of the lactoferrin piglets, which revealed that oral lactoferrin contributed to increasing the absorption efficiency of iron in the small intestine. In addition, the iron levels of piglets (hemoglobin and tissue iron) on day 8 were higher than those on day 21 probably because iron is consumed in the piglets to maintain essential physiological activities. Therefore, we believed that lactoferrin supplement could enhance the iron level in suckling piglets via increasing the iron absorption.

Iron absorption mainly occurs in the duodenum of suckling piglets, where iron is absorbed by enterocytes via the divalent metal transporter 1 (DMT1) pathway in mammals [24]. The absorbed iron is stored in the ferrin and exported to the systemic circulation by ferroportin (FPN), and then bound to iron transferrin to be transported to the site where it is needed [25]. Hence, DMT1 and FPN are the major iron transporters for intestinal iron absorption. Previous investigation revealed that the up-regulated gene expressions of DMT1 and FPN indicate the increased need for dietary iron to meet body demands [26]. In our present study, the protein expression of DMT1 was down-regulated in the LF piglets compared with the CON piglets on day 8, suggesting that the iron demand of the LF piglets was lower than that of the CON piglets. These results were consistent with our present results showing that higher concentrations of serum hemoglobin and hepatic iron were detected in the LF piglets. Interestingly, the protein expression of FPN was also up-regulated in the LF piglets compared with the CON piglets. The up-regulation of FPN protein expression showed that more iron was released from the small intestine (mainly duodenum) to the systemic circulation, which provided more iron for body erythropoiesis and limiting iron toxicity by avoiding iron accumulation in the intestine [27]. Therefore, we believe that the up-regulation of FPN protein expression may be positively associated with the higher concentration of the dendunal iron in the LF piglets. The expression of FPN is also highly linked to the hepcidin expression, which plays a crucial role in regulating systemic iron homeostasis [28,29]. Low production of hepcidin causes systemic iron overload, whereas excess expression of hepcidin leads to the anaemia symptoms. However, in the current results, there was no significant difference of the expression of hepcidin in the serum and liver among all groups, implying that hepcidin did not participate in the regulation of iron status of suckling piglets. Furthermore, the gene expression of lactoferrin receptor (LFR) was higher in the LF piglets than those in the CON piglets on day 8 in the present study. Up-regulated gene expression of LFR implied that lactoferrin participated in the iron absorption and LFR may be involved in iron absorption via an unique way, as the lactoferrin can be the iron transport through a LFR-mediated process [30,31]. Therefore, those data suggested that lactoferrin supplement enhanced the iron absorption of suckling piglets by up-regulating LFR gene expression.

Oxidative stress, which influences the modification of proteins, lipid oxidation and other metabolisms, is induced by the imbalance between oxidation and antioxidation correlation. A single high dose of FeDex injection can generate hydroxyl radicals that lead to oxidative stress [5]. Therefore, we measured the antioxidant capacity of the duodenum and serum to understand the effect of combined iron injection and lactoferrin on body health. It is known that GSH-px and SOD are the vital components of antioxidant system, and T-AOC is the indicator of the endogenous antioxidative capability [32]. In the current study, we provided the evidence that the serum T-AOC duodenal GSH-px activities were significantly higher in the LF piglets than those in the CON piglets, and the duodenal SOD activity was statistically increased in the in the LF piglets than that in the CON piglets on day 21. The increased activities of GSH-px and T-AOC improved the suckling piglets’ ability to resist oxidative stress and inflammation, as GSH-px and SOD contributed to the inhibition of production of reactive oxygen species (ROS), which are the major causes of oxidative stress [33,34]. In agreement with our present results, Wang et al. also proved that supplementation with bovine lactoferrin (feed dosage: 2.5 g bLF/kg body weight) up-regulated the mRNA expressions of SOD and CAT, and the activity levels of GSH-px and SOD in the piglets [35]. These combined results suggested that lactoferrin supplement effectively improved the antioxidant ability and reduced the risk of oxidative stress caused by injection of FeDex in suckling piglets.

The immune system is particularly sensitive to oxidative stress, as ROS damage the function of membrane lipids and the control of signal transduction of gene expression the in immune cell [36,37]. The immune response is regulated by a complex interplay among the anti-inflammatory cytokines and pro-inflammatory cytokines, therefore, the serum cytokines are the direct indicators to evaluate the immune status of animal [38]. IL-10 is an anti-inflammatory cytokine that plays a vital role in the immune response. In the present study, the serum concentration of IL-10 secreted by Th2 cells was statistically higher in the LF piglets on day 8 than that in the CON piglets, indicating that the LF piglets had a stronger innate immune response to the challenge. Furthermore, the serum concentrations of TNF-α and IL-1β secreted by Th1 cells were lower in the LF piglets than those in the CON piglets. Similarly, Donovan et al. demonstrated that dietary bovine lactoferrin appeared to stimulate a balanced Th1/Th2 cytokines immune response, and showed a tendency for immune cells to secrete anti-inflammatory cytokines (IL-10) and reduce the secretion of pro-inflammatory cytokines (TNF-α and IL-1β) [39]. Overall, the lactoferrin supplement could strengthen the adaptive immunity by increasing the IL-10 level and decreasing TNF-α and IL-1β levels.

## 5. Conclusions

In summary, our results provide the evidence that a combination of oral lactoferrin and iron injection is an effective method to improve the iron level via up-regulating the expression of the LFR gene, enhance the antioxidant abilities and modulate the cytokines activities in the suckling piglets. In further study, we will investigate the effect of lactoferrin concentration on weaned piglets.

## Figures and Tables

**Table 1:** Primers used for real-time quantitative PCR analysis of *Dcytb, TF, TFR1, TFR2, LFR* and *β-actin*.

Gene	Primer Sequence (5′-3′)	Accession Number
*Dcytb*	Forward: TCCACGCAGGGTTGAATAC	*NM_001128452·1*
	Reverse: GCCCAAGGAAGCAGAAAGAC	
*TF*	Forward: GTATCCGCAGAAAACACCG	*NM_001244653*
	Reverse: AGGACAGGCACCAGACCAC	
*TFR 1*	Forward: CAGTTGAACAGAATGGCACG	*NM_214001*
	Reverse: CAGACTCAGACCCATCTCCCT	
*TFR 2*	Forward: CAGACTCAGACCCATCTCCCT	*XM_003124374*
	Reverse: CCTCCCAGTCTCCCCTTAT	
*LFR*	Forward: GTGGGYGATCGCTGGTCCA	*NM_017625*
	Reverse: CCTCCTCCACCRATGCAGTG	
*β-actin*	Forward: ATGCTTCTAGACGGACTGCG	*XM_003357928.4*
	Reverse: GTTTCAGGAGGCTGGCATGA	

**Table 2:** Effects of lactoferrin on serum iron parameters of suckling piglets ^1^.

Items	Day 8		Day 21		SEM ^2^	*p*-Values		
	CON	LF	CON	LF		Diet	Age	Diet × Age
Hemoglobin, g/L	91.01 ^a^	105.12 ^b^	71.65 ^c^	77.22 ^c^	6.89	0.041	<0.001	0.045
Serum Iron, μmol/L	31.49	42.35	28.44	33.54	2.68	0.075	0.169	0.124
TIBC ^3^, μmol/L	134.98	138.96	123.76	139.27	12.35	0.551	0.451	0.651

^1. a,b,c^ Means within a row with different superscripts are different at *p* < 0.05. Values are means of 6 replicates per treatment; CON, control; LF, Lactoferrin; ^2^ SEM, standard error of mean. ^3^ Total iron binding capacity.

**Table 3:** Effects of lactoferrin on the iron concentrations in the tissues and digesta of suckling piglets ^1^.

Items	Day 8		Day 21		SEM ^2^	*p*-Values		
	CON	LF	CON	LF		Diet	Age	Diet × Age
Liver, mg/kg	253.94	269.68	173.99	214.45	36.51	0.039	0.298	0.215
Spleen, mg/kg	318.72	299.68	149.47	131.42	52.03	0.732	0.005	0.732
Kindey, mg/kg	40.75	65.22	35.87	33.66	2.68	0.215	<0.001	0.175
Muscle, mg/kg	13.59	14.21	8.56	9.30	1.68	0.418	<0.001	0.433
Dudenum, mg/kg	2.84	4.47	1.38	1.41	0.61	0.188	0.001	0.201
Jejunum, mg/kg	2.25	1.64	1.15	0.90	0.32	0.197	0.010	0.577
Ileum, mg/kg	2.51	2.04	0.88	1.81	0.45	0.137	0.052	0.615
Digesta ^3^, mg/kg	1.06	0.65	1.83	1.16	0.11	<0.001	<0.001	0.217

^1^ Values are means of 6 replicates per treatment; CON, control; LF, Lactoferrin; ^2^ SEM, standard error of mean; ^3^ Digesta was collected in the terminal ileum of suckling piglets.

**Table 4:** Effects of lactoferrin on serum antioxidant parameters of suckling piglets ^1^.

Items ^2^	Day 8		Day 21		SEM ^3^	*p*-Values		
	CON	LF	CON	LF		Diet	Age	Diet × Age
SOD, U/mL	75.85	79.82	72.26	80.25	10.55	0.711	0.919	0.903
T-AOC, U/mL	11.07 ^a^	9.03 ^a^	3.01 ^b^	13.05 ^a^	1.75	0.032	0.253	0.003
MDA, U/mL	1.95	2.64	4.13	2.84	0.84	0.737	0.167	0.260
GSH-px, U/mL	467.69	753.84	823.07	1012.82	103.09	0.048	0.037	0.709

^1. a,b^ Means within a row with different superscripts are different at *p* < 0.05. Values are means of 6 replicates per treatment; control (CON); Lactoferrin (LF); Superoxide dismutase (SOD ^2^); Total antioxidant capacity (T-AOC); Malondialdehyde (MDA); Glutathione peroxidase (GSH-px); standard error of mean (SEM ^3^).

**Table 5:** Effects of lactoferrin on duodenal antioxidant parameters of suckling piglets ^1^.

Items ^2^	Day 8		Day 21		SEM ^3^	*p*-Values		
	CON	LF	CON	LF		Diet	Age	Diet × Age
SOD, U/mg protein	39.53 ^a,b^	36.73 ^a,b^	33.59 ^a^	47.53 ^b^	5.09	0.136	0.508	0.030
T-AOC, U/mg protein	0.71 ^a^	0.54 ^a,b^	0.30 ^b^	0.51 ^a,b^	0.11	0.784	0.020	0.039
MDA, U/mg protein	0.98	0.84	0.52	0.37	0.09	0.140	<0.001	0.950
GSH-px, U/mg protein	76.13	79.09	76.07	127.98	12.96	0.038	0.088	0.087

^1. a,b^ Means within a row with different superscripts are different at *p* < 0.05. Values are means of 6 replicates per treatment; control (CON); Lactoferrin (LF); Superoxide dismutase (SOD ^2^); Total antioxidant capacity (T-AOC); Malondialdehyde (MDA); Glutathione peroxidase (GSH-px); standard error of mean (SEM ^3^).
